# Clinical factors influencing residual subcutaneous tissue after skin-sparing and nipple-sparing mastectomy with immediate breast reconstruction

**DOI:** 10.3389/fonc.2025.1516479

**Published:** 2025-02-26

**Authors:** Menekse Turna, Hale Basak Caglar

**Affiliations:** Department of Radiation Oncology, Anadolu Medical Center, Kocaeli, Türkiye

**Keywords:** residual fibroglandular tissue, skin-sparing mastectomy, nipple-sparing mastectomy, breast reconstruction, postoperative radiotherapy

## Abstract

**Background:**

Skin-sparing mastectomy (SSM) and nipple-sparing mastectomy (NSM) have emerged as increasingly preferred alternatives to traditional mastectomy, largely due to their enhanced cosmetic outcomes and elevated levels of patient satisfaction. Nonetheless, the oncological safety and implications associated with residual breast tissue in these surgical procedures continue to raise significant concerns. The objective of this study is to evaluate the influence of various clinical and surgical factors on residual subcutaneous tissue in patients undergoing SSM and NSM.

**Methods:**

This retrospective cohort study encompassed breast cancer patients who underwent postoperative radiotherapy following SSM and NSM with immediate breast reconstruction from November 2020 to April 2024. Clinical and demographic data, including age, tumor size, axillary staging, molecular subtype, genetic analysis, and surgical details, were systematically collected. Additionally, radiation treatment planning CT scans were assessed to measure residual subcutaneous tissue thickness at multiple anatomical regions. The correlation between residual subcutaneous tissue thickness and clinical factors was subsequently analyzed.

**Results:**

The median age was 45 years (range, 31-61). Among the total patients, 20 underwent SSM (52.63%), and 18 underwent NSM (47.37%). An acceptable residual subcutaneous tissue distance (≤5 mm) was observed in 21 breasts (55.26%), while 17 breasts (44.74%) did not meet this criterion. Analysis demonstrated a statistically significant but modest positive correlation between RFT thickness and age (r = 0.38, p = 0.02), minimal positive correlation was observed between RFT thickness and clinical tumor size (r = 0.08, p = 0.042). A significant effect of contralateral breast surgery on residual subcutaneous tissue thickness was noted (F = 8.38, p < 0.001). Additionally, the results also revealed a statistically significant inverse correlation between RFT thickness and axillary involvement (r = -0.18, p = 0.005), suggesting that thicker flaps are associated with reduced axillary involvement. There was no significant difference in RFT thickness between NSM and SSM groups (Chi² = 0.47, p = 0.491).

**Conclusion:**

A significant proportion of patients undergoing SSM and NSM exhibit residual subcutaneous tissue thickness that exceeds acceptable limits, which may vary based on clinical and pathological factors. Further research involving larger cohorts and prospective designs is essential to identify additional contributing factors and optimize indications for postoperative radiotherapy.

## Introduction

Breast cancer surgery has evolved significantly over the years, with the primary goals being to achieve optimal cosmetic and functional outcomes without compromising oncological safety (1–3). Although breast-conserving approaches are commonly practiced, mastectomy may still be preferred for certain patients, especially with the growing popularity of risk-reducing surgeries facilitated by advancements in genetic analysis (4–6). Despite improvements in early detection and evidence supporting breast-conserving treatments, more radical surgical procedures are necessary for specific cases (7–9). Traditionally, these situations required a modified radical mastectomy (MRM) (10). However, less radical mastectomies, such as skin-sparing mastectomy (SSM) and nipple-sparing mastectomy (NSM), have recently been proposed as alternatives for selected patients (11–14).

SSM and NSM are preferred over total mastectomy due to their superior cosmetic outcomes and higher patient satisfaction (11, 13, 14). However, the oncological safety of these procedures remains a topic of ongoing evaluation, as they tend to leave more residual breast tissue compared to traditional mastectomy techniques (12, 15–17). Retrospective studies have demonstrated that local recurrence rates after SSM are similar to those after MRM, but the clinical implications of this residual breast tissue are unclear (18, 19). Residual breast tissue can remain, depending on the type of surgery, the surgeon, and their experience, even in total mastectomy specimens (20–22). This residual tissue is considered a potential cause of recurrence after mastectomy, particularly in the form of locoregional recurrence involving the skin or subcutaneous tissue (22).

This study aims to evaluate the thickness of residual subcutaneous fibroglandular tissue (RFT) after SSM and NSM among patients receiving radiotherapy and to identify the clinical and surgical factors that influence this thickness. The goal is to determine which patients are at higher risk for residual tissue, based on their clinical or surgical characteristics.

## Materials and methods

We conducted a retrospective cohort study involving breast cancer patients who received postoperative radiation therapy following SSM or NSM with immediate breast reconstruction between November 2020 and April 2024. Data were collected from institutional medical records, which included patient demographics, clinical history, treatment details, and imaging data. All procedures were conducted in accordance with relevant legal and institutional guidelines, with informed consent obtained from all participants.

### Study population and selection criteria

The study included breast cancer patients who underwent either SSM or NSM followed by immediate breast reconstruction and subsequently received postoperative radiation therapy. The inclusion and exclusion criteria were defined as follows:

### Inclusion criteria

- Female patients diagnosed with breast cancer.
- Patients undergoing SSM or NSM with immediate breast reconstruction.

### Exclusion criteria

- Patients who underwent simple mastectomy or modified radical mastectomy.
- Patients with tissue expanders instead of implants.
- Patients without breast reconstruction.
- Patients who developed skin flap necrosis post-surgery.

Patient demographics and clinical characteristics were collected through a retrospective review of medical records, including age, weight, body mass index (BMI), initial tumor size, tumor size at surgery, axillary staging, reason for surgery, contralateral breast surgery, molecular subtype, genetic analysis history, genetic mutations, family history, operation center, tumor location, comorbidities, and neoadjuvant chemotherapy.

### Data collection

The radiation treatment planning CT scans were retrospectively evaluated. CT images were acquired with patients immobilized in the supine position with their arms above their heads, using a vacuum bed with a slice thickness of 2.5 mm (Discovery RT, General Electric (GE) Healthcare, Milwaukee, WI, USA). Coronal, axial, and sagittal slices were analyzed in three dimensions to measure the distance from the skin to the prosthetic tissue.

Two radiation oncologists with expertise in breast radiotherapy conducted simultaneous perpendicular measurements from the breast prosthesis to the skin at a ninety-degree angle to assess the thickness of the RFT. Measurements were taken from various regions, including the medial, lateral, central, and infraclavicular areas of the prosthesis, as well as the axillary tail and inframammary fold for each breast. The largest value for each patient was recorded. All measurements were performed using the Eclipse (version 15.5, Varian Medical Systems) treatment planning system software. A RFT thickness of 5 mm or less was considered acceptable, in line with literature standards (22).

### Statistics

Demographic variables were summarized using descriptive statistics. Differences between groups were assessed using Pearson’s chi-square, Fisher’s exact, Kruskal-Wallis, and Wilcoxon rank-sum tests. For comparisons between skin-sparing and nipple-sparing mastectomy, chi-square tests and Student’s t-tests were employed. Correlations were analyzed using Pearson for age, BMI, weight, and clinical tumor size, and Kendall’s Tau for residual axillary involvement. ANOVA was used to evaluate the impact of contralateral breast surgery on skin flap thickness. All analyses were conducted using IBM SPSS Statistics (Version 21) with a significance threshold of p < 0.05.

## Results

The study included 37 patients and 38 breasts, with a median age of 45 years (range, 31–61 years). Among these, 20 patients (52.63%) underwent SSM, while 18 patients (47.37%) underwent NSM. Of the cohort, 14 patients (36.8%) underwent surgery performed by different surgeons across various centers, whereas 24 patients (63.2%) were treated at our institution by three surgeons.

**Figure 1:**
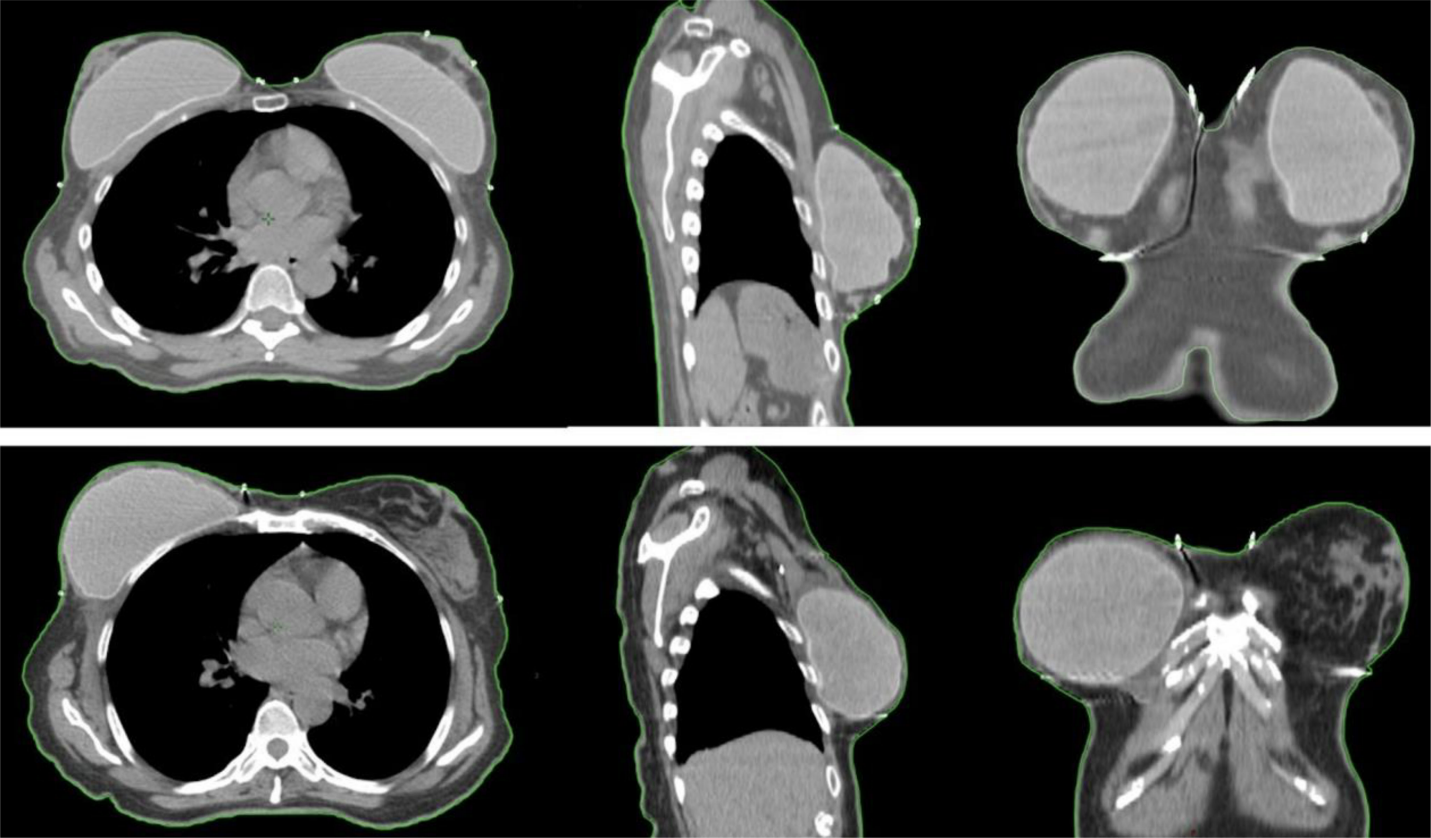
Examples of two patients with acceptable and non-acceptable RFT. The upper images illustrate a case deemed non-acceptable in the left breast, where the residual subcutaneous tissue thickness exceeds 5 mm, depicted in sagittal, coronal, and axial planes. The lower images demonstrate an acceptable case in the right breast, with a RFT thickness of less than 5 mm, also shown in sagittal, coronal, and axial planes.

**Figure 2:**
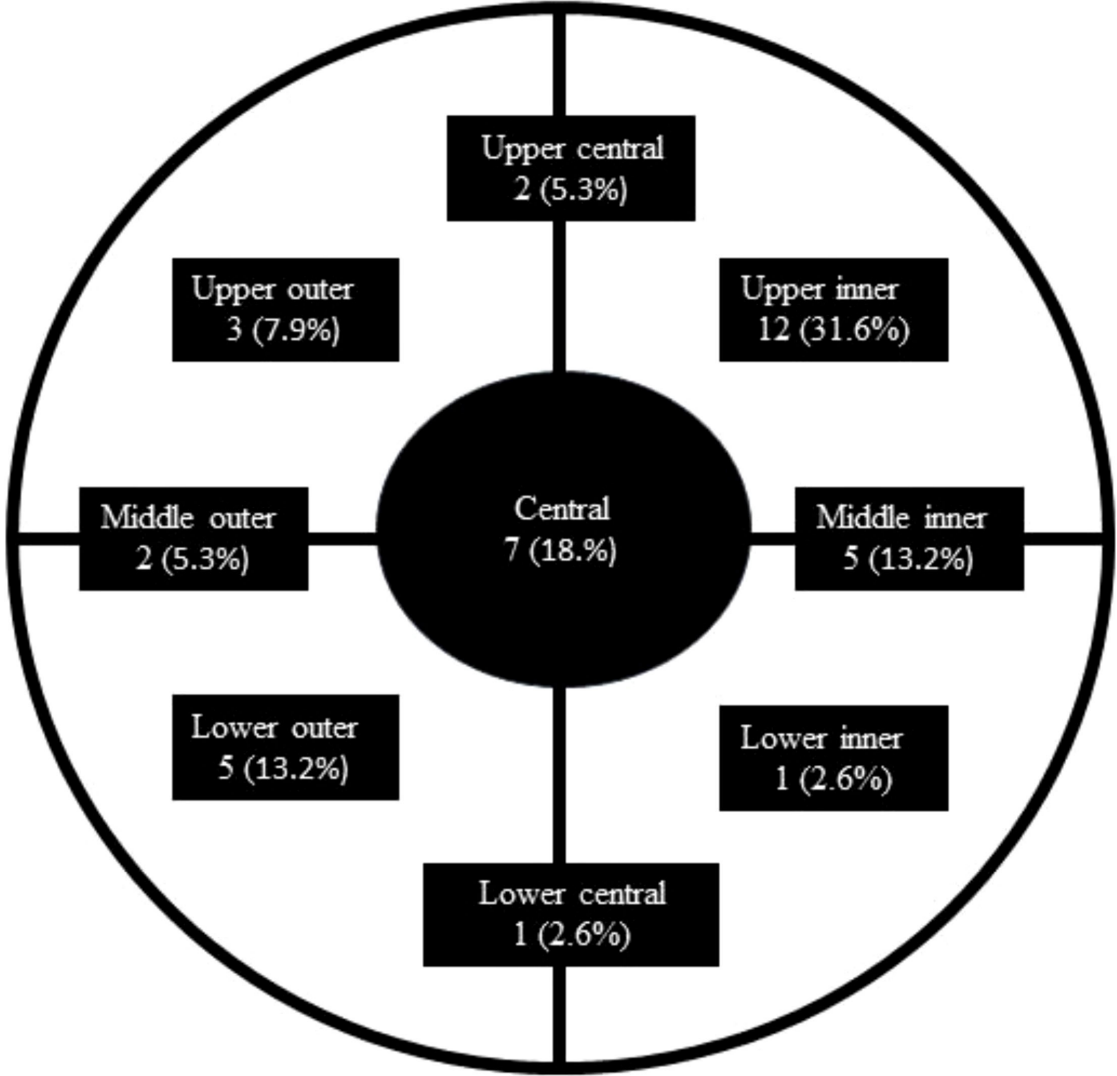
Anatomical representation showing the distribution of the greatest RFT within the right breast.

Patient, tumor, and treatment characteristics are summarized in **Table 1**. The mean clinical tumor size was 35.84 mm (range, 10–100 mm), while the mean pathological tumor size at surgery was 19.92 mm (range, 0–70 mm). The median weight was 67 kg (range, 46–90 kg), and the median BMI was 25.01 (range, 19.14–33.91). The mean RFT thickness was 6.0 ± 4.7 mm. Acceptable RFT thickness (≤5 mm) was achieved in 21 breasts (55.26%), while 17 breasts (44.74%) exceeded this threshold (**Figure 1**). The greatest residual RFT was observed in the upper inner region (12, 37.5%), followed by the central region (7, 21.9%). The distribution in other regions is summarized in **Figure 2**.

**Table 1:** Patient, tumor and treatment characteristics.

Patient/Tumor Factors	n (%)
Lesion Number	
Multicentric	18 (47.37%)
Unifocal	20 (52.63%)
Laterality	
Right	21 (55.26%)
Left	17 (44.74%)
Contralateral Breast Surgery	
No Surgery	18 (47.37%)
NSM	11 (28.95%)
SSM	7 (18.42%)
Breast conserving surgery	2 (5.26%)
Pathology	
Invasive Ductal Carcinoma	34 (89.47%)
Invasive Lobular Carcinoma	4 (10.53%)
Tumor Subtypes	
Hormone Positive	21 (55.26%)
HER2 and Hormone positive	4 (10.53%)
HER2 type	6 (15.79%)
Triple negative	7 (18.42%)
Treatment Sequance	
Neoadjuvant	22 (57.89%)
Upfront Surgery	16 (42.11%)
Comorbidities	
Absent	26 (68.42%)
Present	12 (31.58%)
Hereditary Gene Analysis	
Present	19 (50%)
Absent	19 (50%)
Detected Pathological mutations*	
Yes	15 (78.94%)
No	4 (21%)
Family History	
Absent	29 (76.32%)
Present	9 (23.68%)

*Only in Hereditary Gene Analysis patients.

Analysis demonstrated a statistically significant but modest positive correlation between RFT thickness and age (r = 0.38, p = 0.02). Similarly, a statistically significant but minimal positive correlation was observed between RFT thickness and clinical tumor size (r = 0.08, p = 0.042). There was no significant difference in RFT thickness between NSM and SSM groups (Chi² = 0.47, p = 0.491). Contralateral breast surgery was found to significantly influence RFT thickness (F = 8.38, p < 0.001), with differing impacts for NSM, SSM, and breast-conserving surgery.

The results also revealed a statistically significant inverse correlation between RFT thickness and axillary involvement (r = -0.18, p = 0.005), indicating that increased RFT thickness is associated with reduced axillary involvement.

No statistically significant associations were identified between RFT thickness and histological subtype, tumor subtype, tumor location, BMI, weight, neoadjuvant versus upfront therapy, surgeon, or mastectomy indication.

## Discussion

In this retrospective study, we evaluated the influence of various clinical factors on residual RFT thickness following SSM and NSM. Our analysis demonstrated that a significant proportion of patients who underwent SSM and NSM had residual skin flap thickness exceeding acceptable thresholds. Younger age, larger tumor size, advanced axillary involvement, and contralateral breast surgery were associated with increased RFT thickness. To our knowledge, this is the first study to systematically evaluate clinical and surgical factors associated with RFT thickness using imaging-based measurements, providing insights into which patients may have been at higher risk for residual tissue.

Residual breast tissue remained depending on the type of surgery and the surgeon’s experience, even in total mastectomy specimens, and was considered a potential cause of recurrence after mastectomy, particularly in the form of locoregional recurrence involving the skin or subcutaneous tissue (20–22). The presence of RFT following SSM and NSM posed significant challenges in clinical decision-making, particularly in determining the optimal adjuvant treatment strategy. This study focused on a high-risk patient group receiving post-mastectomy radiotherapy, where the presence of RFT may have held less clinical significance. However, in intermediate- or low-risk patients who did not routinely require radiotherapy after mastectomies, RFT could have emerged as a potential parameter influencing post-mastectomy radiotherapy decisions.

Existing literature highlighted variability in surgical and radiotherapy practices regarding acceptable residual tissue and radiotherapy decisions (22). Radiation oncologists often made decisions regarding radiotherapy after these surgeries in a manner similar to classic mastectomies (23). In a multidisciplinary international survey of post-operative radiotherapy practices, radiation oncologists expressed uncertainty about the oncologically acceptable amount of residual tissue after SSM and NSM, whereas most breast surgeons indicated that a cut-off of 1 mm to 5 mm was the most appropriate (23). Moreover, radiation oncologists were twice as likely to recommend radiotherapy compared to surgeons for cases with large residual skin flaps (23).

An oncologically safe reference value of 5 mm for skin flap thickness had been reported. Residual breast tissue was observed in 59.5% of patients after SSM in a series of 42 patients, with a residual disease rate of 9.5% in those with skin flaps thicker than 5 mm (24). These findings were independent of factors such as age, BMI, stage, and breast volume. According to the recommendations of the German Society of Radiation Oncology (DEGRO), post-mastectomy radiotherapy following SSM or NSM should have been considered for patients with classic risk factors (25). Additionally, premenopausal women with thick subcutaneous tissue and residual breast tissue greater than 5 mm were also recommended for postoperative radiotherapy (25).

Our study’s findings of a high prevalence of RFT highlighted the importance of understanding residual breast tissue following SSM and NSM. Although routine imaging was not performed in our clinic, evidence from a multidisciplinary international survey of post-operative radiotherapy practices indicated that only a minority of respondents consistently requested breast imaging after SSM and NSM to assess residual tissue. Despite the absence of high-level evidence, some authors advocated for the evaluation of residual breast tissue using imaging after these procedures, suggesting that post-mastectomy radiotherapy should have been considered when residual tissue was present (23).

For patients who already had an indication for post-mastectomy radiotherapy due to preoperative factors, the presence of RFT may not have been a significant concern. In such cases, leaving a thicker flap could potentially have reduced complication rates, particularly the risk of skin necrosis, which was less common with thicker flaps (26, 27). However, for early-stage breast cancer patients who would not otherwise have required radiotherapy but were considered for adjuvant radiotherapy due to residual tissue after NSM or SSM, the issue became more critical. In these cases, irradiating the prosthesis could have resulted in deformation, leading to unfavorable cosmetic outcomes (28, 29). The decision to leave a thicker flap should have been carefully considered to balance the reduction of complications like skin necrosis with the potential for long-term cosmetic issues due to prosthesis deformation.

Patients undergoing prophylactic mastectomy tended to have a higher incidence of residual breast tissue compared to those undergoing therapeutic NSM (22). In our series, all patients underwent surgery for invasive cancer, with indications such as multicentric tumors, large tumor size, high tumor-to-breast ratios, or pathological genetic mutations. Therefore, the findings from our study may not have been directly applicable to patients undergoing prophylactic mastectomy.

The study had several limitations, including the small sample size, its retrospective design, and the limited representation of patients managed by each surgeon. There was no high-level evidence from studies demonstrating an increased risk of recurrence associated with RFT thickness exceeding 5 mm. The 5 mm cutoff used in this study lacked robust validation from high-quality research. Moreover, computed tomography may not have been the most reliable imaging modality for assessing RFT thickness, as magnetic resonance imaging was generally regarded as a more precise and superior technique for this evaluation. Our patient cohort represented a high-risk population requiring radiotherapy following SSM and NSM, and these findings may not have been generalizable to residual breast tissue outcomes in risk-reducing procedures or in patients with earlier-stage invasive cancer or carcinoma *in situ*. Additionally, data on tumor-to-nipple distance and tumor-to-skin distance were unavailable due to the lack of preoperative imaging for all patients.

## Conclusion

Our study highlighted that a significant proportion of patients undergoing SSM and NSM had residual skin flap thickness exceeding acceptable thresholds, influenced by various clinical and pathological factors. While we did not recommend routine post-operative imaging for all patients, given the implications of residual tissue on recurrence risk and adjuvant treatment strategies, we aimed to emphasize that high-risk groups may have benefited from post-operative breast imaging. Future research involving larger, prospective cohorts is essential to further investigate the factors contributing to residual tissue formation and refine radiotherapy indications, ultimately enhancing treatment outcomes and patient care.

## Data Availability

The raw data supporting the conclusions of this article will be made available by the authors, without undue reservation.
